# Diagnostic accuracy of prehospital serum S100B and GFAP in patients with mild traumatic brain injury: a prospective observational multicenter cohort study – “the PreTBI I study”

**DOI:** 10.1186/s13049-021-00891-5

**Published:** 2021-06-02

**Authors:** Sophie-Charlott Seidenfaden, Julie Linding Kjerulff, Niels Juul, Hans Kirkegaard, Mette Fogh Møller, Anna-Marie Bloch Münster, Morten Thingemann Bøtker

**Affiliations:** 1 Research and Development, Prehospital Emergency Medical Services, Central Denmark Region, Olof Palmes Allé 34 2, Aarhus, Denmark; 2 Department of Clinical Medicine, Aarhus University, Incuba Skejby, bld. 2, Palle Juul-Jensens Blvd 82, Aarhus, Denmark; 3 Department of Anaesthesiology and Intensive Care, Section North, Neurointensive Care Unit, Aarhus University Hospital, Palle Jull-Jensens Blvd. 161, Aarhus, Denmark; 4 Research Centre for Emergency Medicine, Aarhus University Hospital, Palle Juul-Jensens Blvd. 99, Aarhus, Denmark; 5 Department of Clinical Biochemistry, Regional Hospital West Jutland, Gl. Landevej 61, Herning, Denmark; 6 Department of Clinical Biochemistry, Hospital of South West Jutland, Finsensgade 35, Esbjerg, Denmark; 7 Department af Anaesthesiology, Regional Hospital Randers, Skovlyvej 15, Randers, Denmark

**Keywords:** Traumatic brain injury, Biomarker, S100B, GFAP, Diagnostic accuracy, Prehospital triage, Emergency medical service

## Abstract

**Background:**

The biomarker serum S100 calcium-binding protein B (S100B) is used in in-hospital triage of adults with mild traumatic brain injury to rule out intracranial lesions. The biomarker glial fibrillary acidic protein (GFAP) is suggested as a potential diagnostic biomarker for traumatic brain injury. The aim of this study was to investigate the diagnostic accuracy of early prehospital S100B and GFAP measurements to rule out intracranial lesions in adult patients with mild traumatic brain injury.

**Methods:**

Prehospital and in-hospital blood samples were drawn from 566 adult patients with mild traumatic brain injury (Glasgow Coma Scale Score 14–15). The index test was S100B and GFAP concentrations. The reference standard was endpoint adjudication of the traumatic intracranial lesion based on medical records. The primary outcome was prehospital sensitivity of S100B in relation to the traumatic intracranial lesion.

**Results:**

Traumatic intracranial lesions were found in 32/566 (5.6%) patients. The sensitivity of S100B > 0.10 μg/L was 100% (95%CI: 89.1;100.0) in prehospital samples and 100% (95% CI 89.1;100.0) in in-hospital samples. The specificity was 15.4% (95%CI: 12.4;18.7) in prehospital samples and 31.5% (27.5;35.6) in in-hospital samples. GFAP was only detected in less than 2% of cases with the assay used.

**Conclusion:**

Early prehospital and in-hospital S100B levels < 0.10 μg/L safely rules out traumatic intracranial lesions in adult patients with mild traumatic brain injury, but specificity is lower with early prehospital sampling than with in-hospital sampling. The very limited cases with values detectable with our assay do not allow conclusions to be draw regarding the diagnostic accuracy of GFAP.

**Trial registration:**

ClinicalTrials.gov identifier: NCT02867137.

**Supplementary Information:**

The online version contains supplementary material available at 10.1186/s13049-021-00891-5.

## Introduction

Trauma to the head potentially results in traumatic brain injury (TBI), but it more often causes a concussion or no brain injury at all [1–3]. This severity spectrum gives rise to a clinical dilemma which causes frequent precautionary hospitalization of mild head trauma patients with resulting upstream crowding and excessive resource consumption in emergency departments (EDs) [4–6].

In ED’s the biomarker serum S100 calcium-binding protein B (S100B) is used as a supportive tool for initial in-hospital triage of adult patients with mild TBI [7, 8]. S100B levels < 0.10 μg/L within 6 h of trauma is considered safe for ruling out traumatic intracranial lesions in adult patients with mild TBI and its use reduces the number of cerebral CTs and lengths of stay in the EDs for patients with mild TBI [8–12]. The biomarker glial fibrillary acidic protein (GFAP) has been proposed as another candidate for triage and rapid risk-stratification in patients with TBI [8, 9, 13], but no cut-point for GFAP has been established.

Implementing a prehospital point-of-care measurement of S100B and/or GFAP may facilitate treating and leaving patients on scene, who have no need for hospitalization. However, no studies have investigated the use of samples drawn that close to the trauma, why release kinetics are unclear regarding safe ruleout of traumatic intracranial lesions from very early blood samples.

The aim of this study was to investigate the diagnostic accuracy of prehospital S100B and GFAP concentrations for ruling out traumatic intracranial lesions in unselected adult patients with mild TBI. We hypothesized that 1) S100B concentrations < 0.10 μg/L in prehospital blood samples from adult patients with mild TBI rules out traumatic intracranial lesions with a sensitivity of > 97%, 2) GFAP from prehospital blood samples rules out traumatic intracranial lesions with a sensitivity of > 97%, but with a lower false positive rate than S100B and, 3) Prehospital S100B and GFAP values yield lower false positive rates than in-hospital values.

## Materials and methods

### Study design

The study (the PreTBI I study, ClinicalTrials.gov identifier: NCT02867137) was an investigator driven, outcome assessor-blinded, prospective, observational, multi-center, diagnostic accuracy, cohort study conducted in the Central Denmark Region from 15 February 2017 to 01 February 2019. The study is reported according to the Standards for Reporting of Diagnostic Accuracy Studies (STARD) guidelines [14].

### Participants and setting

**Fig. 1:**
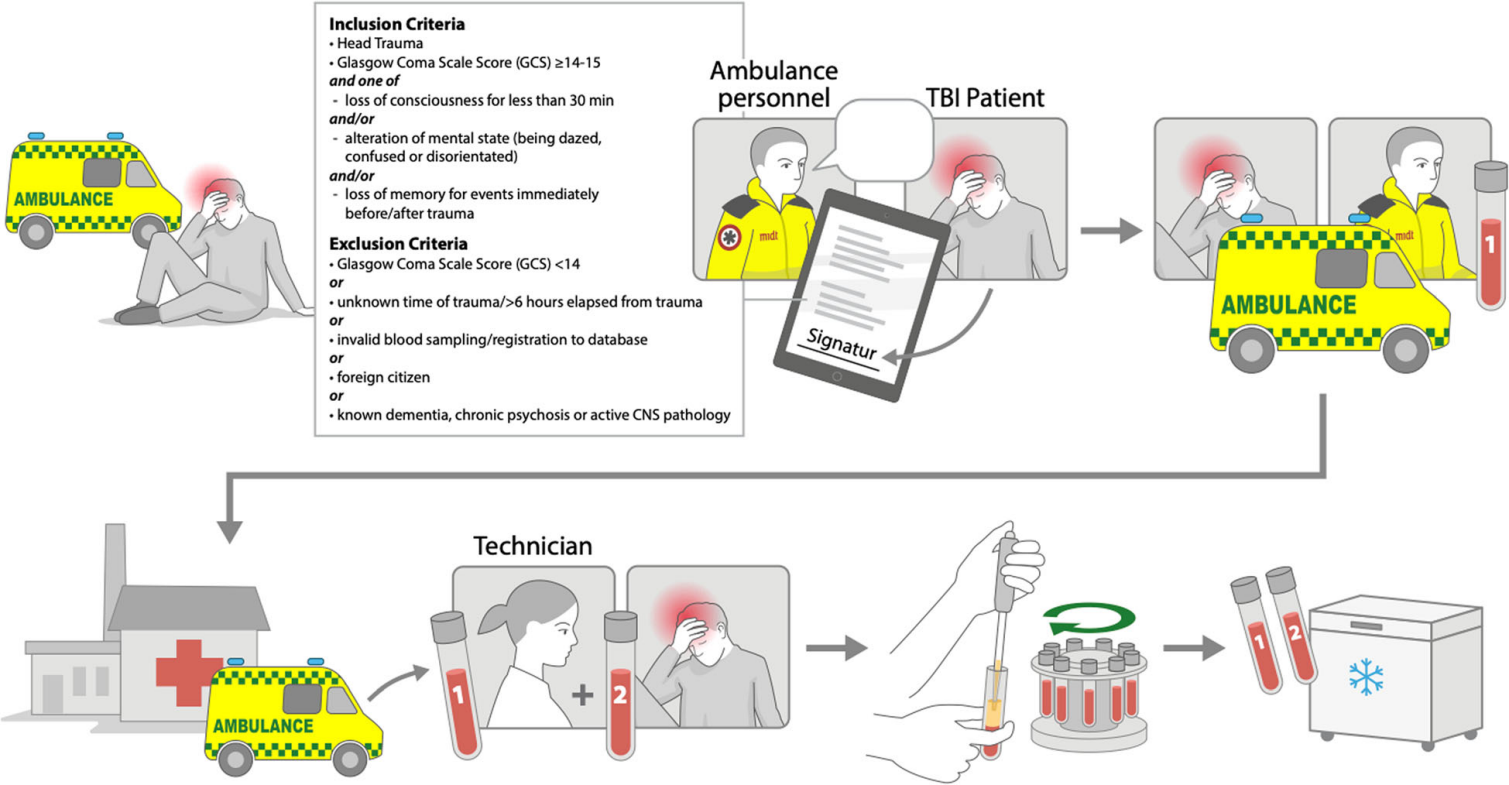
Enrolment procedure. Enrolment procedure of patients with mild TBI with listed inclusion and exclusion criteria, informed consent and blood sampling procedures by prehospital ambulance personnel and in-hospital laboratory technicians in the setting of the PreTBI I study

Eligible patients were adults ≥18 years of age who had suffered head trauma and was transferred by an ambulance by the Prehospital Emergency Medical Services, Central Denmark Region within 6 h of trauma. The patient inclusion and exclusion criteria are listed in Fig. 1.

The Central Denmark Region is one of five Danish regions. The region is inhabited by 1.3 million people, accounting for 23% of the total Danish population. The Danish National Health Service is a tax-supported system, ambulance services are subsidized, and all ambulance dispatch in the region is coordinated through one Emergency Medical Coordination Center (EMCC). Ambulance personnel have access to consultation with critical care physicians staffing the EMCC and rapid-response vehicles. Ambulance personnel are obligated to consult with a critical care physician if they wish to leave a patient on scene. Five centers with emergency facilities participated in the study: four regional hospitals (West Jutland, Randers, Viborg and Horsens) and one university hospital (Aarhus).

Ambulance personnel selected patients for enrolment in the study at the scene of the accident. Ambulance personnel were trained to suspect TBI based on standard operating procedures. Oral and written informed consent was obtained from all participants prior to inclusion. A tablet device with a direct online connection to the PreTBI Database, provided by TrialPartner® (Lucidity, Dept. of Clinical Medicine, Aarhus University, Denmark), allowed for electronic participant signature of informed consent forms and collection of prehospital clinical data (Fig. 1).

Following patient inclusion, prehospital blood samples were collected from the routinely inserted peripheral venous catheter either on scene or en route to hospital. Patients were subsequently transferred to one of five emergency hospitals, where in-hospital blood samples were collected, alongside routine diagnostic workup and treatment (Fig. 1).

### Index test and biochemical analysis

The index tests were S100B and GFAP concentrations in prehospital blood samples. The prehospital blood sample was drawn from a peripheral venous catheter into a 5.5 mL adaptable Sarstedt Monovette® (Sarstedt AG & Co., Nümbrecht, Germany) for serum.

The prehospital blood sample was transported at ambient temperature by ambulance and passed to the laboratory technician at the hospital during collection of the in-hospital sample (Fig. 1). The in-hospital blood sample was drawn into a 6 mL Becton Dickinson® (Becton, Dickinson and Company, New Jersey, USA) serum tube, along with routine samples collected as part of the clinical workup upon ED visits. The in-hospital blood sample was handled under standardized preanalytical conditions (kept at room temperature for 30 min until centrifuged). All blood samples were marked with timestamps and unique research identification numbers.

All samples were centrifuged at 2200 G for 10 min within four hours of collection, and serum was aliquoted into four 1 mL CryoPure (Sarstedt AG & Co., Nümbrecht, Germany) vials containing 0.5–0.6 mL serum and stored at − 80 °C.

All biochemical analysis was performed at Department of Clinical Biochemistry, Regional Hospital West Jutland, Herning. Samples were analyzed in blinded batches within 6 months of collection.

Serum S100B concentrations were measured with a routine Cobas S100 chemiluminescence immunoassay using a Cobas® e602 analyzer (Roche Diagnostics, Mannheim, Germany) after one freeze-thaw cycle.

Serum GFAP concentration was measured after one freeze-thaw cycle using a commercial Human GFAP enzyme-linked immunosorbent assay (ELISA), according to the manufacturer’s protocol (BioVendor, Brno, Czech Republic) for research only. Standard curves and results were generated using a Multiskan FC Microplate Photometer, software version 1.00.96 (Thermo Fisher Scientific, Vantaa, Finland). All samples were assayed in duplicate, and average results were used for analysis. According to the manufacturer, the lower limit of detection for this assay was 0.045 ng/mL, and the lower limit for quantification was set to 0.50 ng/mL. All concentrations lower than the limit of detection were reported as zero, and all concentrations between the limit of detection and 0.50 ng/mL were reported as 0.50 ng/mL.

A precondition for S100B concentrations to be diagnostically useful is a hemolysis index < 10 [15]. Interference indices (Hemolysis, Icterus, and Lipemia) were generated for all samples using the saline protocol on an Alinity® c analyzer (Abbott Diagnostics, Illinois, USA) after two freeze-thaw cycles. Only the hemolysis index results were included in the data analysis.

Clinical information and reference standard results were not available to performers of the index tests.

### Reference standard

The reference standard was endpoint adjudication of the target-condition traumatic intracranial lesion, based on medical records. An outcome assessor committee, consisting of two senior researchers (NJ, MTB), evaluated all patient courses at the end of the study period. The target-condition traumatic intracranial lesion comprised an abnormal cerebral CT examination and/or neurosurgical observation/intervention and/or death caused by TBI within 7 days of trauma. Cerebral CT examinations were categorized as normal or abnormal with respect to the type of traumatic intracranial hemorrhage (subdural, epidural, subarachnoid, and intracerebral hemorrhage), cerebral edema, pneumocephalus, cerebral contusion, and/or skull cap/base fractures. Neurosurgical observation/intervention was registered as a dichotomized yes/no-option based on clinical information on admission to a neurointensive care unit and/or neurosurgery performed due to TBI. Death within 7 days due to TBI was assessed by identification of all patients dying within seven days and reviewing causes of death registered in the Cause of Death Register, in addition to medical records.

The outcome assessors were blinded to all S100B and GFAP values. Cases of discrepancy were resolved by discussion and consensus. Cases of inconclusive cerebral CT examinations were consulted with a specialist in neuroradiology.

### Variables and outcome measures

The predefined test positivity cut-off was a S100B level ≥ 0.10 μg/L, yielding two result categories. Cut-off was determined from the 2014 Scandinavian Neurotrauma Committee guideline [7]. The predefined cut-off for GFAP was any detectable concentration above detection limit of the assay at ≥0.045 ng/mL. The primary outcome was sensitivity of S100B in relation to rule out of traumatic intracranial lesion in patients with mild TBI from prehospital blood samples. Secondary outcomes were the remaining diagnostic accuracy measures for S100B and GFAP values in prehospital and in-hospital samples. Tables of secondary outcomes are presented in Additional file 1. We also performed a post-hoc sensitivity analysis of diagnostic accuracy to account for a hypothetical maximal effect of prehospital transport on pre-analytical factors as previously evaluated in a prior study [16]. Methodology and results concerning this post-hoc sensitivity analysis is presented in the Additional file 2.

### Data sources

All patients underwent prehospital clinical assessment, and ambulance personnel registered initial prehospital data in the PreTBI Database. The personnel were guided through the inclusion and consent procedures by a simple tablet algorithm (Fig. 1). Prior to initiating the study, ambulance personnel received information and education about tablet and algorithm use.

Ambulance personnel registered initial GCS, time of trauma, isolated head trauma or multi trauma, suspected alcohol and/or narcotic intake, known antithrombotic treatment, vomiting, loss of consciousness, neurological deficits, seizures, and clinical signs of cranial fractures. The first set of prehospital vital parameters was extracted from the electronical real-time prehospital medical record. Relevant medical history, prescription medication, trauma mechanism, alcohol consumption, cerebral CT examinations, neurosurgical interventions, intensive care unit admission, and final diagnosis were extracted from the electronical in-hospital medical records.

Demographic data, vital status, comorbidities for Charlson Comorbidity Index score calculation, and cause of death was extracted from the Danish National Patient Registry, the Danish Causes of Death Register and the Central Person Registry administered by the Danish Health Data Authority.

### Study size

We predefined that we wanted the capability to demonstrate that a traumatic intracranial lesion can be ruled out with a negative predictive value of 99% (95%CI: 95;100) with an S100B < 0.10 μg/L. The sample size calculation was conducted by Biostatistical Advisory Service, Faculty of Health, Aarhus University, based on the following preconditions: 30% of TBI patients have S100B below guideline cut-off < 0.10 μg/L and 7% have a traumatic intracranial lesion. Our study required 500 patients with two-tailed alfa = 0.05 and 1-beta = 0.90. Inclusion was performed as consecutive sampling and ended at sample size saturation.

### Statistical methods

All statistical analysis was done using STATA© intercooled, version 15, (StataCorp LP, College Station, Texas, USA). Missing data were not imputed. Categorical data are presented as numbers and proportions. Continuous data are presented as means with 95% confidence interval (CI) or medians and interquartile ranges (IQR) according to distribution. Normality was assessed by visual inspection of histograms and QQ-plots. The S100B and GFAP concentrations were log-transformed to meet linearity prior to analysis. Diagnostic accuracy was estimated in two-by-two tables and are presented as percentages with 95% CI.

For comparison between groups, a parametric paired t-test of log-transformed variables and non-parametric Wilcoxon signed rank test of ordinary variables were conducted or confidence intervals were evaluated. All calculations were two-sided and *p*-values < 0.05 were considered statistically significant.

### Ethics

Approvals were obtained from the Danish Data Protection Agency (journal no. 1–10–72-379-16), the Regional Scientific Ethical Committee System (journal no. 1–10–72-107-16) and the Danish Patient Safety Authority (journal no. 3–3013-2505/1) prior to conduction of the study. The study was reported to ClinicalTrials.gov before inclusion of the first patient (identifier: NCT02867137). The study was performed in accordance with the Declaration of Helsinki. Participants were not exposed to additional invasive procedures or alteration of treatment.

The biomarker values in study samples were not disclosed to the treating medical staff and thus not used for clinical decision-making. Participants did not experience any physical or physiological advantage or disadvantage by participating in the study. Participation in the study did not cause patient or system delays.

## Results

### Participant characteristics

**Fig. 2:**
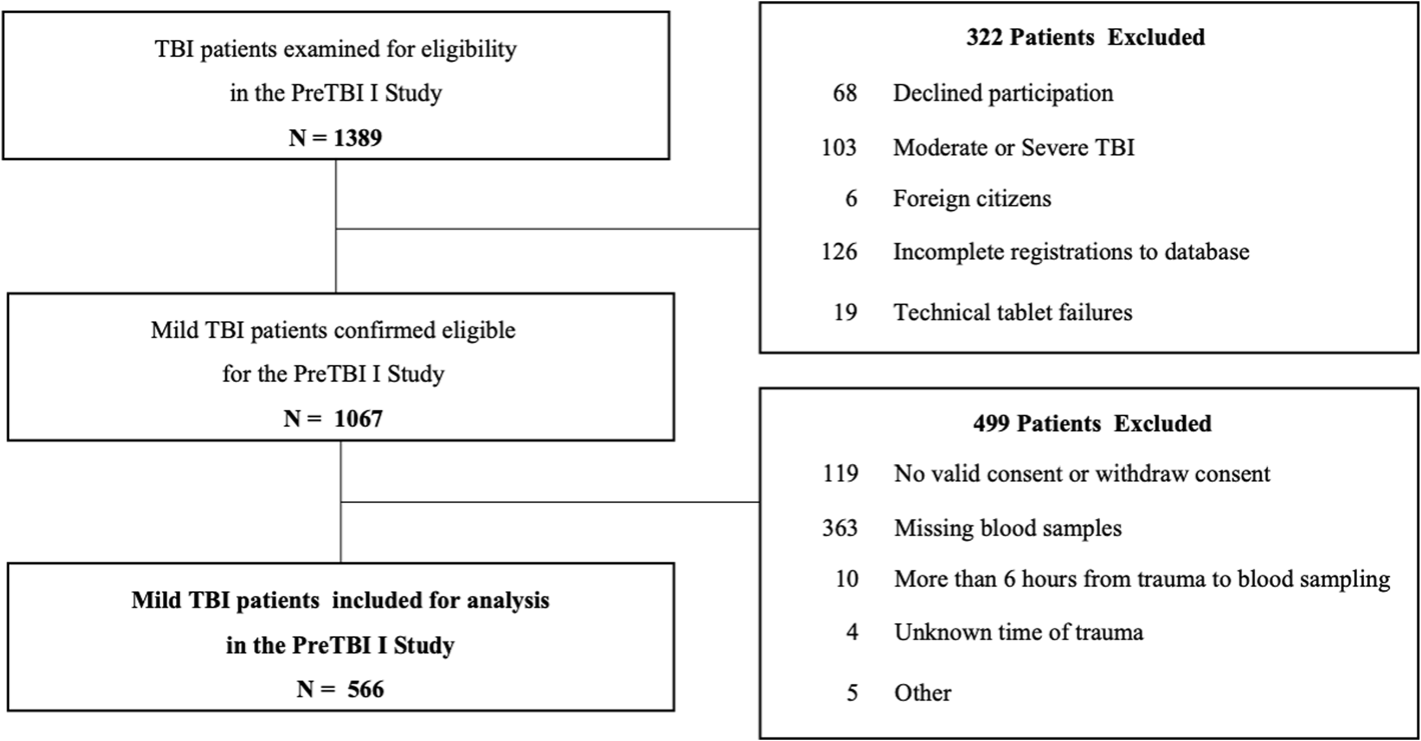
Patient Inclusion. Flow diagram of patient inclusion and exclusion in the PreTBI I study

During the two-year study period, 1389 patients were examined for eligibility in the study. Of 1067 eligible patients, 566 (53.0%) adult patients with mild TBI were included (Fig. 2).

Table 1 shows the overall baseline patient characteristics. The initial GCS was 15 in 453/566 (80.0%) of the included patients, and 113/566 (20.0%) presented with GCS 14. The main trauma mechanism was a fall from under 2 m (62.8%). Antiplatelet/anticoagulant treatment was registered in medical records of 144/556 (25.9%) of patients. Alcohol consumption prior to trauma was suspected in 161/521 (30.9%) patients.

**Table 1:** Baseline characteristics of included mild TBI patients *N* = 566

Variable	n/Valid Cases (%)
**Sex and Age**	
Female, n/N (%)	237/566 (41.9)
Age years, median (IQR)	62 (45;74)
**Charlson Comorbidity Index**	
0, n/N (%)	161/566 (28.4)
1–2, n/N (%)	138/566 (24.4)
3–4, n/N (%)	159/566 (28.1)
≥ 5, n/N (%)	108/566 (19.1)
**Antiplatelet/−coagulant treatment**	
Yes, n/N (%)	144/556 (25.9)
- Acetylsalicylic acid, n/N (%)	65/144 (45.1)
- ADP-receptor antagonist, n/N (%)	32/144 (22.2)
- Vitamin K antagonist, n/N (%)	18/144 (12.5)
- NOAC, n/N (%)	32/144 (22.2)
No, n/N (%)	412/556 (74.1)
**Trauma Mechanism**	
Traffic, n/N (%)	143/557 (25.6)
Fall < 2 m, n/N (%)	350/557 (62.8)
Fall > 2 m, n/N (%)	17/557 (3.1)
Violence, n/N (%)	14/557 (2.5)
Other, n/N (%)	33/557 (5.9)
**Alcohol/drugs prior to trauma**	
Suspected, n/N (%)	161/521 (30.9)
Not suspected, n/N (%)	360/521 (69.1)
**GCS at inclusion**	
GCS 15	453/566 (80.0)
GCS 14	113/566 (20.0)

### Biomarker concentrations

The median time from trauma to sampling was 45 min (IQR; 27;84) for prehospital samples and 108 min (IQR; 85;149) for in-hospital samples (*p*-value < 0.001) (Table 2).

**Table 2:** Sampling time and biomarker concentrations in prehospital and in-hospital blood samples from mild TBI patients

	Prehospital serum samples	In-hospital serum samples	***P***-value
Time from trauma to sampling min., median (IQR)	45 (27;84)	108 (85;149)	< 0.001*
S100B μg/L, mean (95%CI)	0.29 (0.26;0.32)	0.17 (0.16;0.18)	< 0.001**
S100B ≥0.10 μg/L, n/N (%)	484/566 (85.5)	398/566 (70.0)	–
S100B < 0.10 μg/L, n/N (%)	82/566 (14.5)	168/566 (30.0)	–
Lowest S100B concentration in patient with intracranial lesion, μg/L	0.13	0.10	–
GFAP ng/mL, range	0.50–1.78	0.50–2.34	–
GFAP, ≥0.045 ng/mL, n/N (%)	6/566 (1.0)	9/566 (1.6)	–
GFAP, < 0.045 ng/mL, n/N (%)	560/566 (99.0)	557/566 (98.4)	–
Hemolysis Index, mean (95%CI)	0.21 (0.20;0.23)	0.054 (0.050;0.058)	< 0.001**

* Continuous, two sample mean, paired, non-parametric Wilcoxon Signed rank test

** Continuous, two sample mean, paired, parametric testing of log-variable

The mean prehospital S100B concentration was 0.29 μg/L (95%CI: 0.26; 0.32) and the mean in-hospital concentration was 0.17 μg/L (95%CI: 0.16; 0.18) (p-value < 0.001). Concentrations were higher in prehospital samples than in in-hospital samples in 512/566 (90.5%) patients.

GFAP concentrations above the detection limit ≥0.045 ng/mL were detected in 6/566 (1.0%) of prehospital and 9/566 (1.6%) of in-hospital samples in a total of 11/566 patients (1.9%). The highest prehospital concentration of GFAP was 1.78 ng/mL and the highest in-hospital concentration was 2.34 ng/mL. GFAP concentrations were higher in prehospital than in-hospital samples in 4/11 (36.4%) patients.

### Clinical patient outcome evaluated by outcome assessors

Initial agreement of the outcome assessors on the target-condition traumatic intracranial lesion was achieved in 535/566 (94.5%) of the cases (Table 3). Cases were discussed in 31/566 (5.5%), and 25/566 (4.4%) had a consensus achieved following discussion, 6/566 (1.0%) after consultation with a specialist in neuroradiology. Cerebral CT examination was performed in 344/566 (60.7%) of the patients. Non-traumatic incidental findings were noted in 5/344 (1.5%) of the cerebral CT examinations. Details on these cases are presented in Additional file 3 (Table 3.1 and 3.2).

**Table 3:** Clinical outcome of mild TBI patients evaluated by outcome assessor committee

Variable	n/Valid Cases (%)
**Traumatic Intracranial Lesions Yes, n/N(%)**	32/566 (5.6)
- Abnormal CTC yes, n/N (%)	32/32 (100.0)
- Neurosurgical Observation or Intervention yes, n/N (%)	4/32 (12.5)
- Death < 7 days secondary to head injury yes, n/N (%)	2/32 (6.25)
**Details on cerebral CT scan**	
Cerebral CT conducted, n/N(%)	344/566 (60.7)
Intracranial Haemorrhage, n/N(%)	24/32 (75.0)
- *Subdural*	*15/24 (62.5)*
- *Epidural*	*0/24 (0.0)*
- *Subarachnoid*	*10/24 (41.6)*
- *Intracerebral*	*3/24 (12.5)*
Cerebral Edema, n/N(%)	1/32 (3.1)
Pneumocephalus, n/N(%)	1/32 (3.1)
Cerebral contusion, n/N(%)	12/32 (37.5)
Skull fracture, n/N(%)	3/32 (9.3)
- *Base*	*2/3 (66.6)*
- *Cap*	*1/3 (33.3)*
- *Facial*	*0/3 (0.0)*
Marshall CT score	
- II	26/32 (86.2)
- III	3/32 (9.4)
- IV	2/32 (6.2)
- V	0/32 (0.0)
- VI	1/32 (3.1)

The target-condition traumatic intracranial lesion was found in 32/566 (5.6%) patients. In these 32 patients, neurosurgical observation and/or intervention was necessary in 4/32 (12.5%) patients, and a total of two patients (6.25%) died within 7 days of their trauma due to TBI. Details on the cases where the cause of death was evaluated are presented in Additional file 3 (Table 3.3).

### Diagnostic accuracy

The sensitivity of S100B ≥0.10 μg/L for an intracranial lesion in prehospital samples was 100% (95% CI: 89.1; 100.0), with a negative predictive value of 100% for a concentration < 0.10 μg/L (95%CI: 95.6; 100) (Table 4). The specificity of prehospital S100B ≥ 0.10 μg/L was 15.4% (95%CI: 12.4; 18.7). The sensitivity of in-hospital S100B ≥0.10 μg/L was 100% (95%CI: 89.1; 100.0), with a negative predictive value of 100% (95% CI: 97.8; 100.0). The specificity of in-hospital S100B ≥ 0.10 μg/L was 31.5% (27.5;35.6) (Table 5).

**Table 4:** Diagnostic accuracy of S100B concentrations in prehospital blood samples for ruling out of traumatic intracranial lesions in mild TBI patients

Prehospital Blood Samples	Intracranial Lesion		
S100B	Yes	No	Total
**≥0.10 μg/L**	32	452	484
**< 0.10 μg/L**	0	82	82
**Total**	32	534	566
**Sensitivity % (95%CI)**	100.0 (89.1;100)		
**Specificity % (95%CI)**	15.4 (12.4;18.7)		
**Positive Predictive Value% (95%CI)**	6.6 (4.6;9.2)		
**Negative Predictive Value % (95%CI)**	100.0 (95.6;100)

**Table 5:** Diagnostic accuracy of S100B concentrations in in-hospital blood samples for ruling out of traumatic intracranial lesions in mild TBI patients

In-hospital Blood Samples	Intracranial Lesion		
S100B	Yes	No	Total
**≥0.10 μg/L**	32	366	398
**< 0.10 μg/L**	0	168	168
**Total**	32	534	566
**Sensitivity (95%CI)**	100.0 (89.1;100.0)		
**Specificity (95%CI)**	31.5 (27.5;35.6)		
**Positive Predictive Value (95%CI)**	8.0 (5.6;11.2)		
**Negative Predictive Value (95%CI)**	100.0 (97.8;100.0)		

The sensitivity of GFAP above the detection limit (0.045 ng/mL) for an intracranial lesion in prehospital samples was 6.2% (95%CI: 0.8; 20.8), with a negative predictive value of 94.6 (95% CI: 92.4; 96.4). (Table 6a, Additional file 1). The specificity of prehospital GFAP concentrations was 99.3% (95% CI: 98.1; 99.8), yielding a false positive rate of 0.007. In-hospital GFAP yielded a false positive rate of 0.009 (Table 6b, Additional file 1).

## Discussion

This investigator driven, assessor-blinded, prospective, observational, multi-center cohort study examined the diagnostic accuracy of prehospital serum S100B and GFAP levels to rule out traumatic intracranial lesions in patients with mild TBI.

We observed a sensitivity of prehospital S100B of 100% for ruling out of traumatic intracranial lesions, which is comparable to the 100% sensitivity of in-hospital samples in this study. More false positives occurred with prehospital S100B sampling than with in-hospital sampling; thus, prehospital samples had a lower specificity of 15.4% compared to 31.5% for in-hospital samples. GFAP concentrations above the detection limit of 0.045 ng/mL were only detected in 1.0% of prehospital and 1.6% of in-hospital samples.

Baseline patient characteristics of the PreTBI I cohort was a median age of 62 years, male predominance, and standing fall accidents as the main cause of accident, and these are consistent with the findings reported in the studies by Peeters et al. [17], Roozenbeek et al. [18], and Brazinova et al. [19].

No other studies have reported the diagnostic accuracy of S100B and GFAP for ruling out traumatic intracranial lesions from prehospital serum samples from patients with mild TBI. Similarly, no studies have yet reported the diagnostic accuracy of blood samples drawn within 1 h of trauma.

Studies by Biberthaler et al. [20]. and Bouvier and colleagues [21] conducted on similar populations of patients with mild TBI were conducted on in-hospital blood samples, but performed early sampling within 3 h of trauma and reported a sensitivity of 99.0 and 100.0%, with specificity of 28 and 30%. A meta-analysis by Undén and Romner [22] reported an in-hospital diagnostic accuracy comparable to the findings in the present study, with pooled sensitivity at 97% (95%CI: 91; 99) and pooled specificity of 40% (95% CI: 30; 51).

The higher S100B levels for prehospital than for in-hospital samples found in the present study are likely caused by differences in the time from trauma to sampling and thus actual kinetic differences between samples, but they may also arise from differences in preanalytical conditions. Use of the hemolysis index as a proxy for sample quality revealed that all samples were much below the clinically accepted value for hemolysis. The difference in hemolysis index between prehospital and in-hospital samples was very small. We have previously examined the effect of simulated prehospital transport by car on S100B values, and we found that transport may increase S100B values by up to 16% (the upper limit of the 95% CI in that study) [16]. Taking this into account, we conducted a post-hoc sensitivity analysis (Additional file 2) to calculate the diagnostic accuracy of S100B when all values were reduced by 16%, assuming an unlikely maximal effect of transport on all samples. The sensitivity of the reduced prehospital S100B concentrations was retained and we thus consider our results to be solid.

Prior to initiating the study, we expected S100B to reach a peak blood concentration within 1–3 h after the trauma [10, 23] and thus we expected prehospital values to be lower than inhospital values. In that case, prehospital sampling could potentially be performed prematurely to peak blood concentrations, and carry a risk of lower sensitivity and higher false negative rates than with inhospital sampling. On the other hand, lower prehospital values could in that scenario also give rise to lower false positive rates as a potential tradeoff. The results demonstrate that prehospital values are higher than in-hospital values and that concentrations peak at or before prehospital blood sampling at a median of 45 min after trauma. Thus, sensitivity with prehospital sampling is as high as with in-hospital sampling, but specificity is lower. Future research should target release kinetics of S100B in very early blood sampling.

The GFAP results of this study was hampered by the detection limit of the chosen GFAP assay at 0.045 ng/mL. When initiating the study, the GFAP assay was chosen to the best of our knowledge at the time. In the meantime, Bazarian et al. suggested a GFAP cut-off at 0.022 ng/ml, why an assay with a lower detection limit should have been used for analysis [24]. On that note, the GFAP results from the low-sensitivity assay used in the current study, will only identify patients with relatively high GFAP concentrations. Only 11 patients had GFAP concentrations above the assay cut-off of 0.045 ng/mL in one of the samples. If these patients had been the most severe cases, these values would have been interesting for triage and identification of high-risk patients. Unfortunately, they were not, and six of these 11 patients did not have an intracranial lesion. As we only observed 1.6% of the samples above detection limit of the assay, we are not able to report valid mean values or conclude anything from the diagnostic accuracy measures of GFAP concentrations in this study. Nevertheless, when cautiously evaluating the results, we find low sensitivity of GFAP as a diagnostic marker for intracranial lesions and that using a cut-off of 0.045 ng/mL for rule-out would yield a high number of false negatives. This is in contrast to findings of previous studies by Bazarian et al., Papa et al. and Welch et al. Bazarian and colleagues concluded that GFAP show high sensitivity and NPV in samples drawn within 12 h of trauma [24–26]. Thus, future investigation of GFAP in very early samples should be performed using an assay with a low detection limit.

To address pitfalls prior to initiation of prehospital sampling, we included unselected patients with mild TBI in this study. Despite 2014 guidelines limitations of age and antiplatelet/anticoagulant treatment, we aimed to test the absolute safety of prehospital S100B measurements as a diagnostic tool, even in cases of uncertainty about age and antiplatelet/anticoagulant treatment. The results of the present study indicate that S100B-based rule out seems safe, even with median age of 62 years and with 25.4% of the cohort receiving antiplatelet/anticoagulant treatment. This conclusion is supported by findings of Thaler et al. [27], who concluded that S100B below 0.10 μg/L accurately predicts normal cerebral CT findings after mild TBI in elderly patients and in patients treated with antiplatelet/anticoagulant treatment.

Based on the findings of this study, pushing forward the in-hospital guideline approach with prehospital point-of-care testing using the same cut-off of 0.10 μg/L will miss no intracranial lesions. Pushing forward the guideline using prehospital point-of-care technology may safely reduce the number of precautionary ED visits by 15%, as long as the patient in question are in no need of other treatment (suturing, other diagnostics i.e.). The 15% reduction must be compared to a current 100% transferal to ED rate to perform in-hospital diagnostics. Ananthaharan et al. [28] and Mikkinen et al. [29] reported that studies validating in-hospital implementation of the 2014 guideline showed poor adherence to the guideline, with a lesser reduction of cerebral CT examination than expected. Hopefully, the results from ultra-early sampling in this present study will increase the confidence to safely discharge mild TBI patients with low S100B concentrations also after early sampling. Introducing point-of-care technology in a rigorous two-step approach, with initial prehospital triage deciding whether the patient needs ED visit based on prehospital concentrations and with in-hospital triage deciding upon CT examination, may be feasible and reduce both ED visits and CT examinations. Whether this approach is cost-effective could be a future research focus.

### Strengths and limitations

A strength to this study was its multicenter design with ambulance personnel from the entire Prehospital Emergency Medical Services, Central Denmark Region including consecutive unselected patients suffering head trauma. The setup was pragmatic and resembles everyday work of ambulance personnel, which strengthens the external validity of the findings. Blinding of outcome assessors is also a strength, as it reduces the risk of detection bias. Lastly, our approach to initial timestamps being the estimated time of trauma rather than time of admission is a strength. This close-to-trauma sampling yields unprecedented insight to applicability of the biomarkers and early diagnostic accuracy.

The relatively high exclusion rate caused by insufficient blood sampling is a limitation of this study. This is explained mostly by lack of timely handover of the prehospital sample and/or forgotten in-hospital sampling and/or invalid marking of samples. These problems were most likely due to a high workload in the EDs, caused by a large hospital fusion process concurrent with the inclusion for this study in 2017–2019. We do not expect this to bias the results of this study.

Using two different types of tubes for blood sampling (Becton-Dickinson and Sarstedt Monovettes) could be considered a limitation. However, we have previously demonstrated no significant difference between S100B concentrations in blood samples drawn in the two types of tubes [16].

A major limitation is the detection limit of the GFAP assay. We only detected GFAP concentrations above the assay detection limit of 0.045 ng/mL in 6 prehospital and 9 in-hospital samples. Patients with mild TBI may have GFAP concentrations below this detection limit and it could be useful for triage.

## Conclusion

Early prehospital and in-hospital S100B levels < 0.10 μg/L can rule out traumatic intracranial lesion following mild TBI with 100% sensitivity in adult patients. The specificity is lower with pre-hospital sampling (15.4%) than in-hospital sampling (31.5%). Early S100B sampling seems safe even when applied to elderly patients and to patients receiving antiplatelet/anticoagulant treatment. Due to a high cut-point of the assay used, we cannot validly conclude on the diagnostic accuracy of GFAP.

## Supplementary Information

**Additional file 1:.** Diagnostic accuracy tables of GFAP concentrations in prehospital blood samples and of GFAP concentrations in in-hospital blood samples for ruling out of traumatic intracranial lesions in mild TBI patients.

**Additional file 2:.** Post-hoc sensitivity analysis of the data in the current study to investigate diagnostic accuracy of prehospital S100B concentrations adjusted for the maximum effect of prehospital sample transport.

**Additional file 3:.** Supplementary clinical patient outcome evaluated by outcome assessors. Cases of cerebral CT examinations marked by one or both assessors as needing evaluation by specialist in neuroradiology, non-traumatic incidental findings on cerebral CT examinations and cause of death within 7 days of the trauma extracted from the Cause of Death Register.

## Data Availability

The data sets used and analysed during the current study is available from the corresponding author upon reasonable request and with relevant permissions from Danish authorities. The data is not publicly available due to protection of personal data.

## Abbreviations

CI: Confidence Interval
CT: Computed Tomography
EMCC: Emergency Medical Call Coordination
ED: Emergency Department
GCS: Glasgow Coma Scale Score
HIL: Hemolysis Icterus Lipidemia
IQR: Interquartile Range
S100B: Serum S100 calcium-binding protein B
GFAP: Serum Glial Fibrillary Acidic Protein
TBI: Traumatic Brain Injury
