# Supplementary material for: Diagnostic accuracy of prehospital serum S100B and GFAP in patients with mild traumatic brain injury: a prospective observational multicenter cohort study – “the PreTBI I study”
Source: Scand J Trauma Resusc Emerg Med. 2021 Jun 2;29:75. doi: 10.1186/s13049-021-00891-5 (PMC8173808; doi:10.1186/s13049-021-00891-5)
Supplement: Supplementary file 1 — Additional file 1:. Diagnostic accuracy tables of GFAP concentrations in prehospital blood samples and of GFAP concentrations in in-hospital blood samples for ruling out of traumatic intracranial lesions in mild TBI patients. [file 13049_2021_891_MOESM1_ESM.docx]

**Additional File 1**

| **Table 6a**  **Legend:** Diagnostic accuracy of GFAP concentrations in prehospital blood samples for ruling out of traumatic intracranial lesions in mild TBI patients | | | |
| --- | --- | --- | --- |
| **Prehospital Blood Samples** | **Intracranial Lesion** | |  |
| **GFAP** | **Yes** | **No** | **Total** |
| ≥**0.045 ng/L** | 2 | 4 | 6 |
| **<0.045 ng/L** | 30 | 530 | 559 |
| **Total** | 32 | 534 | 566 |
| **Sensitivity % (95%CI)** | 6.2 (0.8;20.8) | | |
| **Specificity % (95%CI)** | 99.3 (98.1;99.8) | | |
| **Positive Predictive Value% (95%CI)** | 33.3 (4.3;77.7) | | |
| **Negative Predictive Value % (95%CI)** | 94.6 (92.4;96.4) | | |

**Diagnostic Accuracy Tables**

| **Table 6b**  **Legend:** Diagnostic accuracy of GFAP concentrations in in-hospital blood samples for ruling out of traumatic intracranial lesions in mild TBI patients | | | |
| --- | --- | --- | --- |
| **In-hospital Blood Samples** | **Intracranial Lesion** | |  |
| **GFAP** | **Yes** | **No** | **Total** |
| ≥**0.045 ng/L** | 4 | 5 | 9 |
| **<0.045 ng/L** | 28 | 529 | 557 |
| **Total** | 32 | 534 | 566 |
| **Sensitivity % (95%CI)** | 12.5 (3.5; 29.0) | | |
| **Specificity % (95%CI)** | 99.1 (97.8; 99.7) | | |
| **Positive Predictive Value% (95%CI)** | 44.4 (13.79;78.8) | | |
| **Negative Predictive Value % (95%CI)** | 95.0 (92.8;96.6) | | |
