# Supplementary material for: Diagnostic accuracy of prehospital serum S100B and GFAP in patients with mild traumatic brain injury: a prospective observational multicenter cohort study – “the PreTBI I study”
Source: Scand J Trauma Resusc Emerg Med. 2021 Jun 2;29:75. doi: 10.1186/s13049-021-00891-5 (PMC8173808; doi:10.1186/s13049-021-00891-5)
Supplement: Supplementary file 2 — Additional file 2:. Post-hoc sensitivity analysis of the data in the current study to investigate diagnostic accuracy of prehospital S100B concentrations adjusted for the maximum effect of prehospital sample transport. [file 13049_2021_891_MOESM2_ESM.docx]

**Additional File 2**

**Post-hoc sensitivity analysis**

*Methods*

Based on knowledge from a previous study on the effect of different preanalytical factors on S100B concentrations, we found that transport may increase S100B concentrations up to 16% (the upper limit of the 95% CI in that study). We performed a sensitivity analysis to the data of the current study to investigate diagnostic accuracy of prehospital S100B concentrations adjusted for the maximum effect of transport. We calculated all diagnostic accuracy measures on the prehospital S100B values after a 16% reduction.

*Results*

The mean of reduced prehospital S100B was 0.24 μg/L (95% CI: 0.22; 0.26). The sensitivity of reduced S100B concentrations in prehospital samples for rule-out of traumatic intracranial lesion was 100% (95% CI: 89.1; 100) with a negative predictive value of 100% (95% CI: 96.9; 100). The specificity of reduced prehospital S100B concentrations was 22.3% (95% CI: 18.8; 26.1) corresponding to false positive rate of 0.78.

| **Table 2.1**  **Legend:** Diagnostic accuracy of 16% reduced S100B concentrations in prehospital blood samples | | | |
| --- | --- | --- | --- |
| **Prehospital blood samples adjusted for effect of transport (16% reduction)** | **Intracranial Lesion** | |  |
| **S100B** | **Yes** | **No** | **Total** |
| **>0.10 ug/L** | 32 | 415 | 447 |
| **<0.10 ug/L** | 0 | 119 | 119 |
| **Total** | 32 | 534 | 566 |
| **Sensitivity (95%CI)** | 100.0 (89.1;100.0) | | |
| **Specificity (95%CI)** | 22.3 (18.8;26.1) | | |
| **Positive Predictive Value (95%CI)** | 7.2 (4.9;10.0) | | |
| **Negative Predictive Value (95%CI)** | 100.0 (96.9;100.0) | | |
