# Supplementary material for: Diagnostic accuracy of prehospital serum S100B and GFAP in patients with mild traumatic brain injury: a prospective observational multicenter cohort study – “the PreTBI I study”
Source: Scand J Trauma Resusc Emerg Med. 2021 Jun 2;29:75. doi: 10.1186/s13049-021-00891-5 (PMC8173808; doi:10.1186/s13049-021-00891-5)
Supplement: Supplementary file 3 — Additional file 3:. Supplementary clinical patient outcome evaluated by outcome assessors. Cases of cerebral CT examinations marked by one or both assessors as needing evaluation by specialist in neuroradiology, non-traumatic incidental findings on cerebral CT examinations and cause of death within 7 days of the trauma extracted from the Cause of Death Register. [file 13049_2021_891_MOESM3_ESM.docx]

**Additional File 3**

**Supplementary clinical patient outcome evaluated by outcome assessors**

| **Table 3.1**  **Legend:** Cerebral CT examinations marked by one or both assessors as needing evaluation by specialist in neuroradiology. | |
| --- | --- |
| **Initial evaluation** | **Final evaluation** |
| 1 case with doubt about whether a small CT attenuation was a cross cut vein or a minor cerebral contusion | Cross cut vein, no cerebral contusion or bleeding (not coded as intracranial lesion) |
| 1 case of suspected minor ventricular bleeding | Colloid cyst, no ventricular bleeding (not coded as intracranial lesion) |
| 2 cases of micro bleeds | In both cases, micro bleeds were chronic and non-traumatic (not coded as intracranial lesion) |
| 1 case of suspected SAH | Confirmed minor SAH (coded as intracranial lesion) |
| 1 case of initial CT examination without traumatic intracranial lesions, but re-admission within 5 days of trauma with SDH | Minor SDH present on initial CT examination (coded as intracranial lesion) |

| **Table 3.2**  **Legend:** Incidental findings on cerebral CT examinations. |
| --- |
| 1 Glioblastoma |
| 1 Meningioma |
| 1 Low grade glioma |
| 1 Ventriculoperitoneal-shunt malfunction |
| 1 Aneurism |

| **Table 3.3:**  **Legend:** Causes of death within 7 days of the trauma extracted from the Cause of Death Register. |
| --- |
| Aortic stenosis (not coded as death due to TBI) |
| Multiple brain bleeds (coded as death due to TBI) |
| Fall, Traumatic Subarachnoid Hemorrhage (coded as death due to TBI) |
| Dissection of abdominal aorta (not coded as death due to TBI) |
| Acute Myocardial Infarction (not coded as death due to TBI) |
| Fall, Heart Failure, Neck fracture (not coded as death due to TBI) |
